# Supplementary material for: Genetic analysis of QTLs controlling allelopathic characteristics in sorghum
Source: PLoS One. 2020 Jul 30;15(7):e0235896. doi: 10.1371/journal.pone.0235896 (PMC7392238; doi:10.1371/journal.pone.0235896)
Supplement: S1 Fig — Circles represent independent runs for each value of J. Each run for J was repeated three times. (PPTX) [file pone.0235896.s001.pptx]

## Slide 1
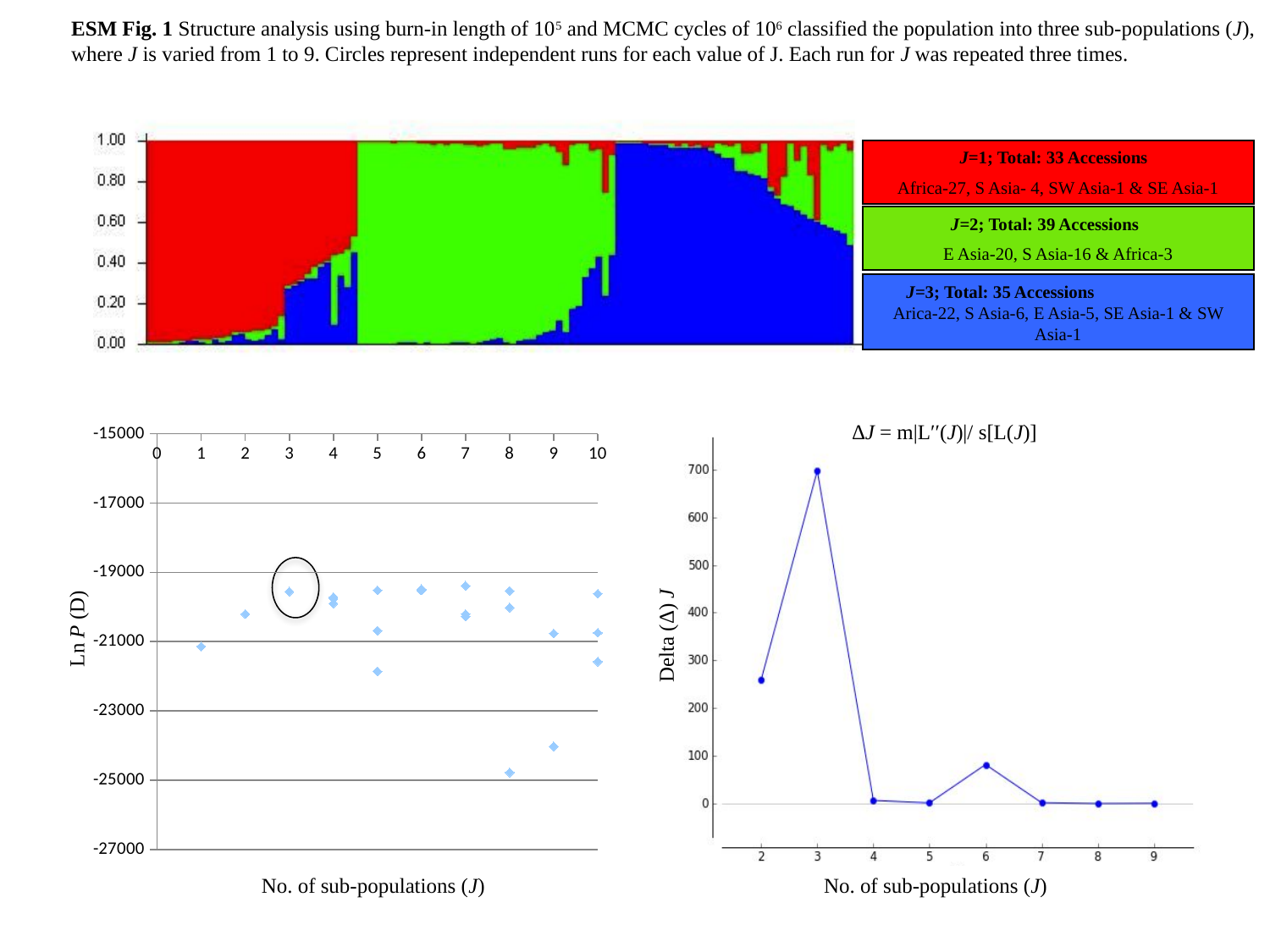

ESM Fig. 1 Structure analysis using burn-in length of 105 and MCMC cycles of 106 classified the population into three sub-populations (J),
where J is varied from 1 to 9. Circles represent independent runs for each value of J. Each run for J was repeated three times.
 J=1; Total: 33 Accessions
Africa-27, S Asia- 4, SW Asia-1 & SE Asia-1
 J=2; Total: 39 Accessions
E Asia-20, S Asia-16 & Africa-3
 J=3; Total: 35 Accessions Arica-22, S Asia-6, E Asia-5, SE Asia-1 & SW Asia-1
### Chart
| Category | |
|---|---|
∆J = m|L′′(J)|/ s[L(J)]
Ln P (D)
Delta (Δ) J
No. of sub-populations (J)
No. of sub-populations (J)
