# Supplementary material for: Genetic analysis of QTLs controlling allelopathic characteristics in sorghum
Source: PLoS One. 2020 Jul 30;15(7):e0235896. doi: 10.1371/journal.pone.0235896 (PMC7392238; doi:10.1371/journal.pone.0235896)
Supplement: S2 Fig — Each cell represents the relationship between two markers with color codes showing the level of significance. (PPTX) [file pone.0235896.s002.pptx]

## Slide 1
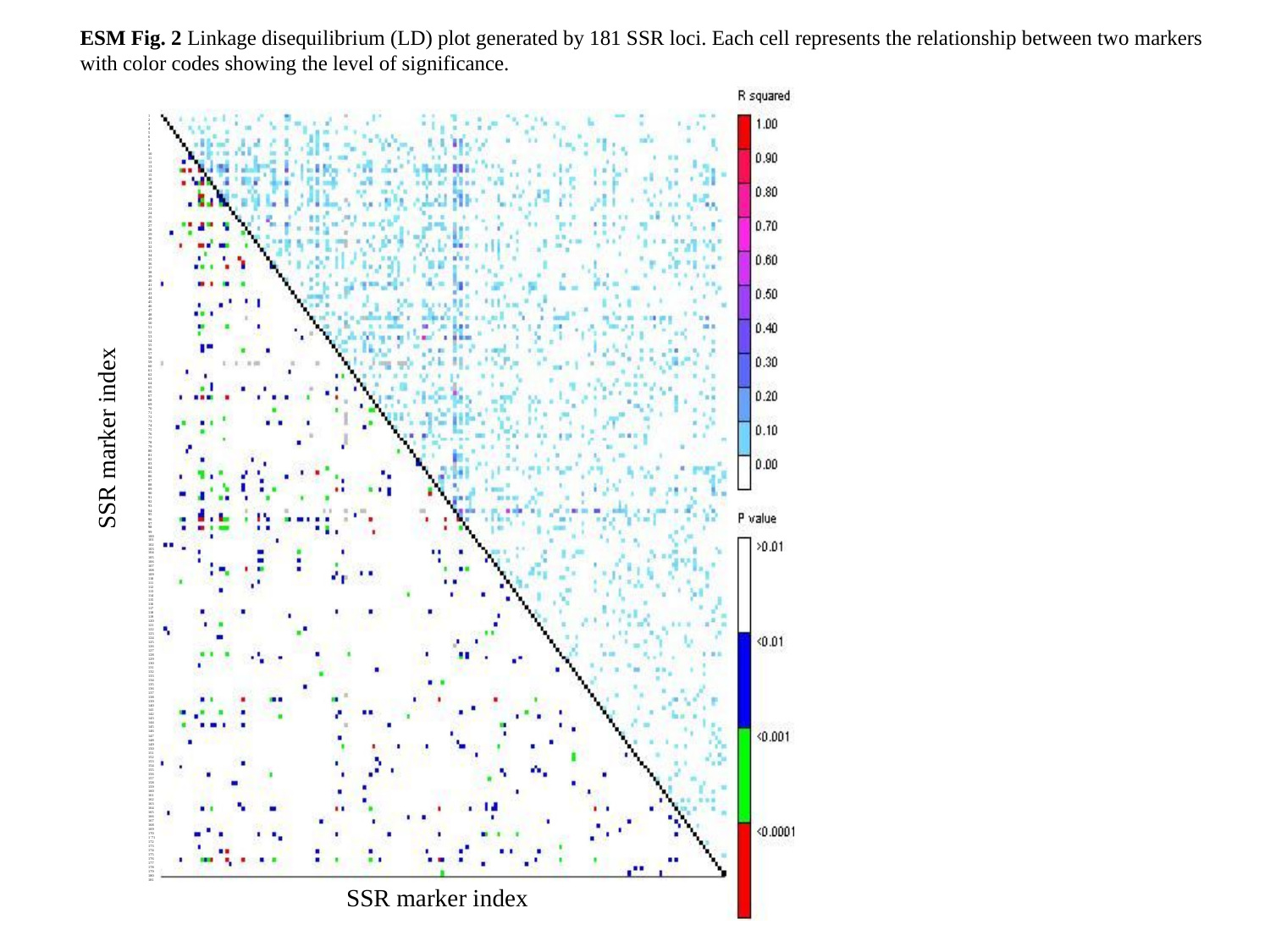

ESM Fig. 2 Linkage disequilibrium (LD) plot generated by 181 SSR loci. Each cell represents the relationship between two markers with color codes showing the level of significance.
1
2
3
4
5
6
7
8
9
10
11
12
13
14
15
16
17
18
19
20
21
22
23
24
25
26
27
28
29
30
31
32
33
34
35
36
37
38
39
40
41
42
43
44
45
46
47
48
49
50
51
52
53
54
55
56
57
58
59
60
61
62
63
64
65
66
67
68
69
70
71
72
73
74
75
76
77
78
79
80
81
82
83
84
85
86
87
88
89
90
91
92
93
94
95
96
97
98
99
100
101
102
103
104
105
106
107
108
109
110
111
112
113
114
115
116
117
118
119
120
121
122
123
124
125
126
127
128
129
130
131
132
133
134
135
136
137
138
139
140
141
142
143
144
145
146
147
148
149
150
151
152
153
154
155
156
157
158
159
160
161
162
163
164
165
166
167
168
169
170
1`71
172
173
174
175
176
177
178
179
180
181
SSR marker index
SSR marker index
