# Supplementary material for: Genetic analysis of QTLs controlling allelopathic characteristics in sorghum
Source: PLoS One. 2020 Jul 30;15(7):e0235896. doi: 10.1371/journal.pone.0235896 (PMC7392238; doi:10.1371/journal.pone.0235896)
Supplement: Attachment — Submitted filename: Review Comments to the Author2.docx [file pone.0235896.s005.docx]

6. Review Comments to the Author

Please use the space provided to explain your answers to the questions above. You may also include additional comments for the author, including concerns about dual publication, research ethics, or publication ethics. (Please upload your review as an attachment if it exceeds 20,000 characters)

Thank you very much for the continuous support and help in revising our manuscript.

Reviewer #1: Authors have partially addressed the comments as they gave answers to the queries raised during the first round but only in their letter not in the main text. Please amend the manuscript based on the suggested changes. For example;

1. Please, add how heritability was calculated, and explain why you refer to SOR1 in the discussion part

Response: Thanks, added in revised manuscript. The method of calculating heritability is added in methods under to heading Statistical Analysis and referring SOR1 in discussion part. Also Table 4. is modified by adding R2 values in new column.

2. Please, add to your methods how the threshold was chosen for QTL and association mapping. Please add R2 and how you calculated "FDR-corrected P value" for the association mapping, that might strengthen your point of using 2 as a threshold.

Response: Thank you very much. We have added the method of FDR-corrected in material and methods and also R2 values for QTLs detected using GWAS and Targetted Association Analysis. The method of FDR corrected P value has been explained in material and methods under heading of Population Structure and association mapping.

3. Please, check the language throughout the manuscript as many sentences are not correct. For example, "Each trait was analyzed with empirical experiment-wise threshold values for significance (P = 0.05) from estimating 1000 permutations"

Response: Thank you very much. The manuscript has been gone through English editing once again by my colleague a native speaker and checked for linguistic errors.

4. In the QTL part, please discuss the QTL co-location as this will support your results.

Response: Thanks, we have already added the one found using several pipelines and added in the results and discussion part.

5. Please rephrase the sentence you added to introduction, “Researchers studying secondary metabolites affecting germination of parasitic weed Striga asicatica (witchweed) first discovered it (Chang et al. 1986). Retrobiosynthetic NMR analysis was ................. ,
materials and methods, "We physically localized the SSR loci that were strongly linked with the traits by BLAST searches of sequences in http://www.phytozome.net/sorghum ……… ",
and to the discussion "also reported an increase in inhibitory effects with increasing concentration of allelochemicals. After statistical analyses, we detected 75% and 100% WSE as optimum --------", as they don’t read well.

Response: Thanks, done.
